# First Approach to the Aroma Characterization of Monovarietal Red Wines Produced from Varieties Better Adapted to Abiotic Stresses

**DOI:** 10.3390/plants12102063

**Published:** 2023-05-22

**Authors:** Francesco Agostinelli, Ilda Caldeira, Jorge M. Ricardo-da-Silva, Miguel Damásio, Ricardo Egipto, José Silvestre

**Affiliations:** 1 Department of Agricultural, Forest and Food Sciences, University of Torino, Via Verdi, 8, 10124 Torino, Italy; francesco.agostinell@edu.unito.it; 2 LEAF—Linking Landscape, Environment, Agriculture and Food, Instituto Superior de Agronomia, Universidade de Lisboa, 1349-017 Lisbon, Portugal; jricardosil@isa.ulisboa.pt; 3 Polo de Inovação de Dois Portos, Instituto Nacional de Investigação Agrária e Veterinária, Quinta de Almoinha, 2565-191 Dois Portos, Portugal; miguel.damasio@iniav.pt (M.D.); ricardo.egipto@iniav.pt (R.E.); jose.silvestre@iniav.pt (J.S.); 4 MED—Mediterranean Institute for Agriculture, Environment and Development and CHANGE—Global Change and Sustainability Institute, Institute for Advanced Studies and Research, Universidade de Évora, Pólo da Mitra, 7006-554 Évora, Portugal

**Keywords:** aroma characterization, climate change, red wines, sensory profile, GC-O, odor active compounds

## Abstract

Adaptation strategies in the wine sector consist of the use of cultural techniques to limit damages caused by climate change, using, among other resources, varieties better adapted to the scenarios of abiotic stress exacerbation, namely water and thermal stress, as well as those more tolerant to heatwaves. With the intention to determine the aromatic characterization of ten monovarietal wines produced from cultivars with high productive performance in a global warming scenario (‘Petit Verdot’, ‘Marselan’, ‘Merlot’, ‘Touriga Franca’, ‘Syrah’, ‘Vinhão’, ‘Bobal’, ‘Preto Martinho’, ‘Trincadeira’, and ‘Alicante Bouschet’), grown in Esporão vineyard (Alentejo, Portugal) and submitted to deficit irrigation (Ks ± 0.5), their aromatic character has been analyzed. Each grape variety was vinified at a small scale, in duplicate, and the wines were evaluated by a sensory panel, which rated several sensory attributes (visual, olfactory, and gustatory). Sensory analysis revealed a discrete appreciation for the monovarietal wines tasted, showing a differentiation at the olfactory level that was not too marked, although present, between the samples. The free volatile compounds were analysed using gas chromatography-olfactometry (GC-O), identified using a gas chromatography-mass spectrometry (GC-MS) technique and semi-quantified using the gas chromatography-flame ionization detector (GC-FID) technique. Based on the interpolation of the results of the various statistical analyses carried out, 49 probable odor active compounds (pOACs) were identified and based on the odor activity values (OAVs), 24 of them were recognized as odor active compounds (OACs) originated mainly during the fermentation processes. An aromatic characterization of the varieties has been proposed.

## 1. Introduction

Wine has accompanied human beings since ancient times [1], and today it is considered one of the most important products originating from the agricultural sector. Fundamental to the appreciation of a wine by the market is its quality, determined by extrinsic factors (label, brand, history of the producer and designation of origin) and by intrinsic factors such as alcohol content, concentration of acids, and the set of factors that determine hedonistic pleasure in the consumer, closely related to sensory sensations [2].

One of the sensory aspects that most interests human beings is olfactory sensations. The complexity of olfactory sensations is often perceived by experts and consumers as a great marker of wine quality, wine typicity and as an intrinsic factor of wine complexity [3,4].

Olfactory perception is related to the presence of different volatile compounds belonging to different chemical families. When it comes to the classification of olfactory molecules present in wines, considering the chemical classes the compounds belong to and their origins among the vinification processes, it is possible to identify four principal classes: (i) grape and varietal volatile odorous compounds, in which are grouped all those compounds coming from the cluster that are normally stored in the cells of the berries, in particular in the cells of the epicarp and in lower concentration also in the cells of the mesocarp, both in free form and as glycosidic, aminoacidic/peptidic precursors; (ii) pre-fermentative volatile odorous compounds, formed during the grape processing that precedes the start of fermentation or formed by thermal, chemical or enzymatic reactions in the must after pressing or during pre-fermentative pellicular maceration; (iii) fermentative volatile odorous compounds, originated by yeasts or bacteria during fermentation processes, such as alcoholic fermentation and malolactic fermentation; and (iv) fermentative/post-fermentative/maturation/aging volatile odorous compounds, a class in which all the aromas developed during wine aging or as a result of the extraction of odorous compounds from wood materials are grouped [5].

The entirety of society, in recent years, has been forced to become aware of the phenomenon that represents, and will represent in the years to come, one of the greatest challenges to the sustainable survival of human beings on planet Earth: Climate Change. The meteorological conditions that may occur during the vegetative-productive season of the vine directly affect its production potential, both in terms of yield and quality [6,7,8,9,10]. 

To reduce the damages caused by climate change in the wine sector, two main strategies can be followed: mitigation (of the factors that cause climate change) and adaptation (of cultural techniques to limit damages caused by the phenomenon in question).

One of the adaptation techniques proposed by researchers that seems to give very promising results, either in the short or medium term, consists in seeking within the grapevine germoplasm those varieties which are better adapted to the scenarios previously described, and which can act as valid substitutes or adjuvants of the varieties currently cultivated [11,12]. 

Following the adaptation technique described, in this article several monovarietal wines were produced from varieties previously selected as better adapted to abiotic stresses, based on the ranking of the varieties according to the water use efficiency, vigor, tolerance to heatwaves, and yield, among other things (https://wineclimadapt.pt/bases-de-dados (accessed on 1 December 2022)). Although the effect of deficit irrigation on the polyphenolic component of the grapes is known (increasing their concentration when this irrigation technique is applied, compared to plain irrigation), not much is known about its effects on the odor components of the grapes and consequently of the wines. Recent studies seem to show that there is a positive correlation between the use of deficit irrigation and the concentration of certain volatile compounds in grapes and wines. In particular, the concentration of esters, terpenes, and C13-norisoprenoids would seem to be raised, compared to plain irrigation, while the concentration of C6-alcohols would seem to undergo a decrease following the application of the above-mentioned technique [8,13].

The ten monovarietal wines that have shown a higher quality potential in the sensorial analysis (‘Petit Verdot’, ‘Marselan’, ‘Merlot’, ‘Touriga Franca’, ‘Syrah’, ‘Vinhão’, ‘Bobal’, ‘Preto Martinho’, ‘Trincadeira’, and ‘Alicante Bouschet’) were chemically screened and aromatically characterized to establish the different aromatic profiles, which will be very useful for the winemakers. 

## 2. Results and Discussion

### 2.1. Basic Monovarietal Wines Chemical Composition

The basic chemical composition of each monovarietal wine is presented in Table 1. All the parameters analyzed show significant differences between the wines. The analysis of the alcoholic strength reveals one of the major problems faced by producers in warm climate zones: the increase in the sugar concentration of the grapes [14,15] and a consequent increase in the alcohol content of the wines, with a maximum value reached of 16.9% vol.; the only exception being the wine obtained from ‘Bobal’ grapes with an alcohol content of 11.6% vol. Such high alcohol contents, in addition to directly influencing the organoleptic characteristics of wines, also determine a stress condition for the yeasts conducting alcoholic fermentation, creating effects on the fermentation kinetics and influencing the success of the fermentation itself [16,17]. The effect of the stressful fermentation environment for the yeasts can be evidenced by both residual sugar (reducing substances) and volatile acidity, which generally shows higher concentrations in samples with higher alcohol content. The analysis of total acidity, fixed acidity, and pH shows results in line with other studies conducted in hot climate regions [18,19].

### 2.2. Sensory Profile

The two replicate wines produced from each variety were submitted to sensory analysis (SA) and the results were analyzed using analysis of variance (ANOVA), principal component analysis (PCA), and hierarchical cluster analysis (HCA), as stated in Section 3.4.6. After grouping the samples according to the classes obtained from the post-hoc differential test of Bonferroni (Table 2), to effectively visualize the existing sensory profile differences, the results of the SA are represented by radars in Table 2. The ANOVA showed that significant differences between the samples did not exist for most attributes assessed by the tasting panel. As can be seen from the radars in Table 2, the samples present a sensory profile shifted more towards the sensory components related to visual and gustatory sensations than to the olfactory ones, with ‘Red Fruit/Berries’ and ‘Cooked Fruits/Jam’ being the most odor discriminating attributes. These sensations could be related to high concentrations of esters and alcohols in the wines; the data are in agreement with other studies conducted in hot environments where the olfactory component of black berry wines is particularly penalized by abiotic stress conditions [20,21].

**Figure 1:**
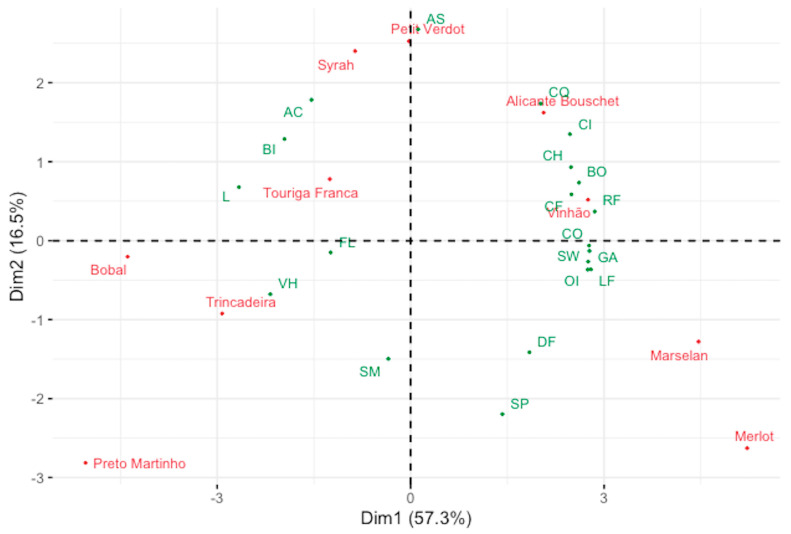
Projection of the wine samples and the sensorial descriptors on the plane defined by the two components of the standardised PCA. [‘L’—‘Limpidity’, ‘CI’—‘Color Intensity’, ‘CQ’—‘Color Quality’, ‘RF’—‘Red fruits/Berries’, ‘DF’—‘Dried Fruits’, ‘CF’—‘Cooked fruits/Jam, ‘VH’—‘Vegetal/Herbaceous’, ‘SP’—‘Spiced’, ‘CH’—‘Chocolate’, ‘SM’—‘Smoke/Toasted’, ‘FL’—‘Floral’, ‘OI’—‘Odor Intensity’, ‘AC’—‘Acidity’, ‘SW’—‘Sweetness’, ‘BI’—‘Bitterness’, ‘AS’—‘Astringency’, ‘BO’—‘Body’, ‘CO’—‘Complexity’, ‘LF’—‘Length/Finish’, ‘GA’—‘General Appreciation’].

**Figure 2:**
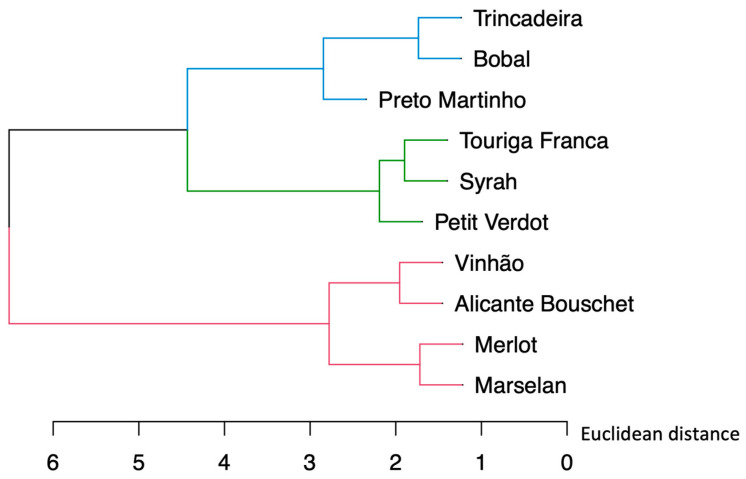
Dendrogram obtained using sensory data results for the monovarietal wine samples.

As an outcome, the grape variety factor had a highly significant effect on the overall quality of the wines, assessed by the ‘Global Appreciation’ attribute, which allowed a great discrimination of the wine samples (Table 2). For a better understanding of the similarities/differences among the samples, the data obtained from the sensory analysis were submitted to the principal component analysis (PCA) and to the hierarchical cluster analysis (HCA) using as observations the monovarietal red wines and as variables the 20 attributes scaled by the panelists. The results are shown, respectively, in Figure 1 and Figure 2. As displayed in Figure 1, the first two dimensions show a cumulative variance of 73.8% with 57.3% from the first component and 16.5% from the second component. The greatest relevance for Component 1 is shown by the variables ‘Red Fruits/Berries’, ‘Length/Finish’, ‘General Appreciation’, ‘Complexity’, ‘Sweetness’, ‘Odor Intensity’, ‘Body’, ‘Cooked Fruits/Jam’, ‘Chocolate’, ‘Color Intensity’, ‘Color Quality’, ‘Bitterness’, ‘Vegetal/Herbaceous’ and ‘Limpidity’, while ‘Astringency’ and ‘Spiced’ show the greatest importance for Component 2.

It is possible to notice that the four monovarietal red wines that have achieved a higher score in the sensory analysis for the ‘General Appreciation’ (‘Vinhão’, ‘Merlot’, ‘Marselan’, and ‘Alicante Bouschet’) are all projected in the positive side of Component 1, where the attributes ‘Color Quality’, ‘Color Intensity’, ‘Chocolate’, ‘Body’, ‘Cooked fruits/Jam’, ‘Red Fruits/Berries’, ‘Complexity’, ‘Sweetness’, ‘Finish’, ‘Odor Intensity’, ‘Dried Fruits’, and ‘Spiced’ are projected, showing a positive correlation among these attributes and the perception of the quality through the panelists. The ‘Petit Verdot’ wine samples show a direct relation with the attribute ‘Astringency’, both being projected on the upper side of the bidimensional space, while the wines produced from the varieties ‘Touriga Franca’ and ‘Syrah’ are more related to the variables ‘Bitterness’ and ‘Acidity’, characterizing these three wine samples as more related to the ‘hard’ part of the taste component. Lastly, the monovarietal red wines produced from the varieties ‘Bobal’, ‘Trincadeira’ and ‘Preto Martinho’ are projected in the space created from the negative side of both axes, where the attributes ‘Vegetal/Herbaceous’, ‘Floral’, and ‘Smoke/Toasted’ are positioned, characterizing these wines with a greener aroma profile. 

**Figure 3:**
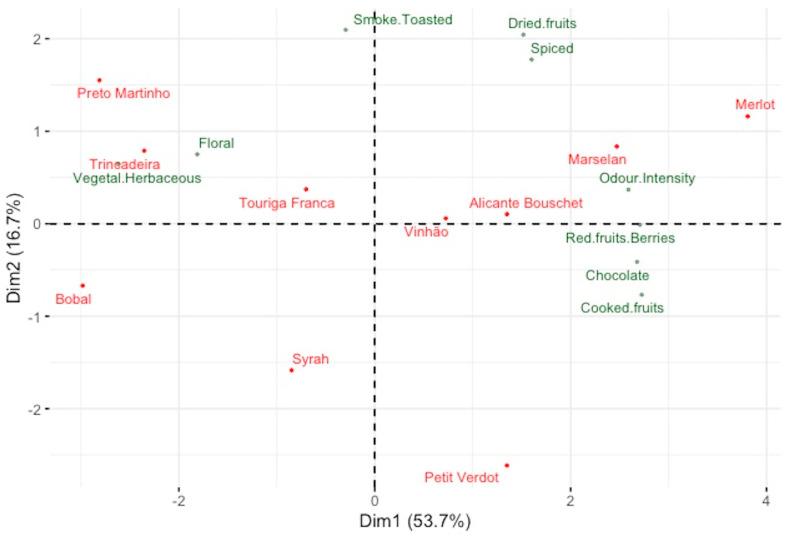
Projection of the wine samples and the sensorial descriptors for aromatic assessment in the plane defined by the two components of the standardized PCA.

The observations extrapolated from the PCA are confirmed with the HCA depicted in Figure 2, where three major groups among the samples have been identified, clustering together the wines as just described. Figure 3 shows a PCA performed considering as variables only the attributes related to aroma evaluation. The first two dimensions show a cumulative variance of 70.4%, with 53.7% from the first component and 16.7% from the second component. This analysis shows a similar distribution of the wine samples to the one presented in Figure 1. It is interesting to highlight that the varietal wines with the highest values in ‘General Appreciation’ (Table 2), namely the wines samples from ‘Vinhão’, ‘Marselan’, ‘Alicante Bouschet’ and ‘Merlot’, are all located on the positive side of Component 1, which are linked with higher intensities of the attributes ‘Odor Intensity’, ‘Cooked Fruits’, ‘Red Fruits’, ‘Chocolate’ and ‘Spiced’ (Figure 3).

### 2.3. Probable Odor Active Compounds Selection and Quantification

The GC-O analysis detected more than 120 volatile compounds. Among them, 49 volatile aromatic compounds were marked by the sniffers as probable odor active compounds (pOACs). To be declared as a pOAC, a compound had to be marked by at least three sniffers. The identification of the compounds was performed by a comparison between chromatograms of the different samples and the spectra obtained by GC-MS of the same sample. 

When possible, the Kovats indices were compared with the one obtained from pure standards of the investigated compounds. At the same time, the odor sensation delivered by them was evaluated. Of the 49 compounds assigned by the sniffers, 37 were identified with confirmation, 6 were identified with probability but could not possibly be confirmed, and 6 remain unknown.

**Figure 4:**
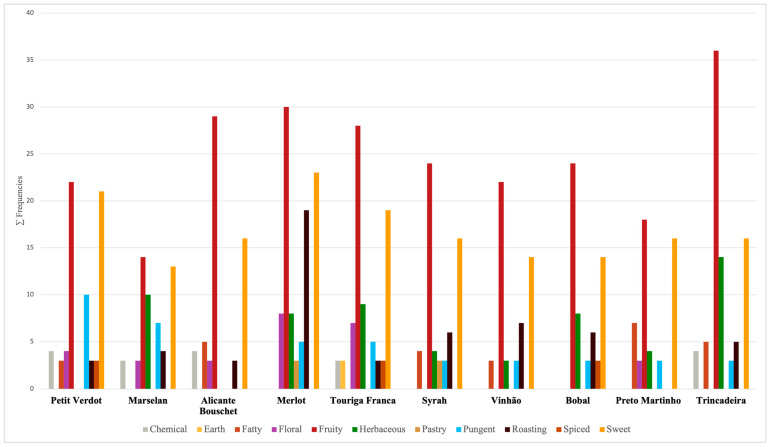
Visualization of the odor profile of the monovarietal red wines obtained analyzing the odors detection frequency in the GC-O analysis and divided by the odor series.

The frequency of detection of the compounds (shown in Table S1) was clustered following the odor series, taking into consideration that the detection frequency can be considered as an estimate of the odors’ intensity [22]. Summing the frequency of identification of each odor series, a visualization of the odors profile of each monovarietal red wine was produced (Figure 4).

The wines present a variable profile in terms of both aromatic sensations and aromatic compounds detected. The prevalent aromatic series among the wines are ‘Fruity’ and ‘Sweet’, sensations determined in the wine samples by acids (e.g., isobutyric acid), alcohols (e.g., 1-butanol and isoamyl alcohols), ketones (e.g., 2,3-butanedione and 2,3-pentanedione) and esters (e.g., ethyl propanoate and ethyl 2-methylbutyrate). Other relevant odor series are ‘Roasting’ (determined by the identification of octanoic acid, isobutanol, 1,2-propanediol, and ethyl hexanoate; and characterizing the ‘Merlot’ wines among the others), ‘Pungent’ (class represented by compounds such as acetic acid and methional, and present with a significant detection frequency in the ‘Petit Verdot’ and ‘Marselan’ wines), and ‘Herbaceous’ (determined by compounds such as propanoic acid, 1,3-propanediol and hexanal, and mainly present in the wines produced from the variety ‘Marselan’, ‘Touriga Franca’, and ‘Trincadeira’). The results obtained from the GC-O partially match with those obtained in the sensory analysis, regarding the olfactory aspects related to the varieties. Moreover, the wines obtained from ‘Merlot’, ‘Syrah’, ‘Trincadeira’, and ‘Alicante Bouschet’, which were found to be the most appreciated varieties during the sensory analysis (Table 2), show a higher frequency of detection of the compounds collected in the aromatic classes ‘Sweet’ and ‘Fruity’ than the other wines. It should be noted that these aromatic classes are representative of wines in which the sensory profile is mainly related to volatile compounds originating during the alcoholic fermentation process [16]. This could be an explanation for the low aromatic differentiation between the varieties that emerged from the sensory analysis; furthermore, this aspect could also be linked to the origin of the grapes where, due to the climatic aspects, all the compounds representative of varietal character could have deteriorated [8,23]. The ‘Herbaceous’ odor series was mostly characteristic of the wines demarked by a lower score in the ‘General Appreciation’, and the same can be said, apart from ‘Merlot’, regarding the odor series ‘Pungent’, and, except for ‘Trincadeira‘ and ‘Alicante Bouschet‘, for the odor series ‘Chemical’. Another interesting correlation between the two analyses can be identified relatively to the ‘Roasting’ aromatic class, mostly detected in the ‘Merlot’ wines, characterized in the sensory analysis by high scores (mostly higher) for the attributes ‘Spiced’, ‘Chocolate’ and ‘Smoke/Toasted’.

#### Quantification of the Probable Odor Active Compounds

**Figure 5:**
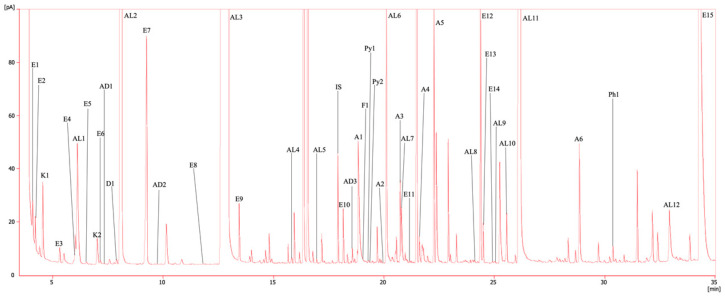
Chromatogram of dichloromethane extract from ‘Syrah’ wine; peak identification in Table 3.

The results of the pOAC quantification (carried out with the method described in GC-FID procedure at Section 3.4.4) are collected in Table 3, while a chromatogram of the ‘Syrah’ wine sample (selected for its representativity of all the varieties) with the identification of the VOCs is shown in Figure 5. An ANOVA was performed on the collected data to detect which compounds showed significant differentiation between the samples. The results, when the analysis of variance detected a significant difference, were divided into classes (indicated by letters placed after the standard deviation) between the various samples.

As can be seen in the Table 3, the chemical class of compounds that recorded a higher concentration compared to the others across all wines is alcohols, with concentrations varying from 730.674 mg/L (‘Vinhão’ wines) to 1914.958 mg/L (‘Bobal’ wines). The second class of compounds recorded with greater concentrations within all the wines is that of esters. Concentrations of these in the analyzed samples were significantly lower than those of alcohols, with total concentrations ranging from 32.942 mg/L (‘Vinhão’ wines) to 59.982 mg/L (‘Marselan’ wines). The acids constitute the third class of compounds in the wines analyzed, with total concentrations ranging from 8.287 mg/L (‘Trincadeira’ wines) to 18.713 mg/L (‘Preto Martinho’ wines). The remaining chemical classes of compounds summed are present in the analyzed samples, with concentrations ranging from 0.942 to 4.206 mg/L. These results are apparently a confirmation of the considerations made following to the analyses previously displayed, again showing wines that, from an aromatic point of view, are mainly characterized by compounds of fermentation origin, with differences between the samples not always so marked as to determine an unambiguous characterization, and with simple sensory profiles linked mainly to volatile compounds that give wines olfactory sensations describable by the adjectives ‘Fruity/Floral/Sweet’ in all samples, ‘Green/Fatty’ in wines produced from grape varieties such as ‘Syrah’, and ‘Touriga Franca’, and wines with a more varied bouquet with notes ranging from spicy to herbaceous, as in the case of ‘Merlot’, ‘Vinhão’ and ‘Marselan’. 

It must be specified that, even without any contact with the wood, some wine samples have shown the presence of the eugenol, which can be classified as a varietal aroma compound in some monovarietal wines [5]. Moreover, in this study, all the wines have shown the presence of at least one of the two methoxypyrazines usually detected and characteristic of French varieties such as ‘Cabernet Sauvignon’, ‘Cabernet Franc’, ‘Carmenère’, and ‘Pinot Noir’ [24].

**Table 3:** Quantification of the probable odor active compounds (pOACs). Data are expressed as mean (mg/L) of the semi-quantification results (among the replicate and repetitions) ± standard deviation. [‘Petit Verdot’ (PV), ‘Touriga Franca’ (TF), ‘Preto Martinho’ (PM), ‘Bobal’ (B), ‘Marselan’ (MA), ‘Alicante Bouschet’ (AB), ‘Syrah’ (SY), ‘Trincadeira’ (TR), ‘Merlot’ (ME), and ‘Vinhão’ (VI)].

Id ^α^	Compound	Odor Descriptors (Threshold in mg/L)	Monovarietal Red Wines									
			PV	MA	AB	ME	SY	TF	VI	B	PM	TR
		Alcohols										
AL1	1-Propanol	Fruity, alcohol, sweet (306) ^δ^	5.076	3.951	5.668	6.213	7.379	4.597	1.655	5.928	4.693	3.776
			±0.702	±1.023	±3.686	±2.914	±1.120	±2.673	±0.034	±0.966	±0.248	±0.527
AL2	Isobutanol *	Cocoa, coffee, banana, cooked potatoes (40.0) ^δ^	68.845 ^b,c^	55.724 ^b,c^	84.300 ^a,b^	45.144 ^b,c^	86.381 ^a,b^	71.435 ^b,c^	36.639 ^c^	125.585 ^a^	119.982 ^a^	59.150 ^b,c^
			±3.570	±12.921	±46.537	±1.246	±2.669	±35.992	±0.039	±12.644	±5.888	±13.514
AL3	Isoamyl alcohols ***	Cocoa, banana, chocolate, pungent, oily (30) ^φ^	646.974 ^c^	560.381 ^c^	737.609 ^b,c^	634.252 ^c^	1179.224 ^a,b^	798.104 ^b,c^	456.168 ^c^	1432.651 ^a^	1192.340 ^a,b^	694.749 ^b,c^
			±45.970	±106.383	±192.191	±11.859	±120.689	±237.967	±0.792	±47.219	±35.060	±93.014
AL4	3-Methyl-1-pentanol ***	Cheese, herbaceous, sour (0.5) ^λ^	0.104 ^b^	0.085 ^b^	0.099 ^b^	0.065 ^b^	0.219 ^a^	0.120 ^b^	0.086 ^b^	0.117 ^b^	0.225 ^a^	0.096 ^b^
			±0.014	±0.020	±0.026	±0.002	±0.017	±0.036	±0.002	±0.019	±0.001	±0.014
AL5	3-Ethoxy-1-propanol	Sweet, tropical fruit (0.1) ^μ^	0.173	0.098	0.137	0.088	0.205	0.064	0.035	0.201	0.100	0.074
			±0.012	±0.031	±0.107	±0.001	±0.013	±0.029	±0.001	±0.022	±0.003	±0.020
AL6	2,3-Butanediol **	Cooked potatoes, soil dust (120) ^δ^	4.443 ^a,b^	7.711 ^a,b^	9.348 ^a^	5.209 ^b^	4.733 ^a^	6.235 ^a,b^	2.091 ^b^	2.601 ^a,b^	6.873 ^a^	3.935 ^a,b^
			±0.456	±1.519	±0.542	±0.171	±1.388	±2.157	±0.161	±1.215	±0.506	±0.524
AL7	1,2-Propanediol **	Toast, sweet (80) ^ρ^	1.746 ^b,c^	1.629 ^a,b^	1.902 ^a^	1.235 ^a,b,c^	1.156 ^a,b,c^	1.374 ^a,b,c^	0.941 ^c^	1.567 ^c^	2.719 ^a,b,c^	0.960 ^b,c^
			±0.214	±0.365	±0.596	±0.025	±0.073	±0.551	±0.002	±0.410	±0.029	±0.141
AL8	1,3-Propanediol **	Green, oily, peppery (N.A.)	0.032 ^a,b^	0.039 ^a,b^	0.046 ^a,b^	nd	0.050 ^b^	0.035 ^a,b^	nd	0.041 ^a,b^	0.051 ^a^	0.042 ^b^
			±0.001	±0.009	±0.001		±0.016	±0.002		±0.007	±0.017	±0.017
AL9	3-Mercaptohexanol **	Herbaceous, oily, not good (0.00006) ^ν^	0.013 ^b^	0.013 ^b^	0.028 ^a,b^	0.012 ^b^	0.050 ^a^	0.026 ^a,b^	0.017 ^b^	0.029 ^a,b^	0.035 ^a,b^	0.021 ^b^
			±0.008	±0.003	±0.004	±0.001	±0.012	±0.001	±0.001	±0.010	±0.001	±0.001
AL10	Benzyl alcohol ***	Flowery, banana, oily (200) ^δ^	0.592 ^b,c^	1.265 ^b^	0.877 ^b,c^	0.804 ^b,c^	2.344 ^a^	0.758 ^b,c^	0.388 ^c^	0.620 ^b,c^	0.715	0.766 ^b,c^
			±0.051	±0.219	±0.153	±0.005	±0.306	±0.200	±0.002	±0.061	±0.026	±0.161
AL11	2-Phenylethanol **	Floral, tobacco, banana (10) ^φ^	216.097 ^a,b,c^	181.421 ^c^	198.238 ^b,c^	218.695 ^a,b,c^	350.514 ^a^	232.402 ^a,b,c^	231.457 ^a,b,c^	341.619 ^a,b^	325.962 ^a,b,c^	192.019 ^c^
			±59.496	±30.282	±9.790	±2.800	±68.624	±35.101	±3.090	±14.156	±0.853	±0.308
AL12	Glycerol *	Roses, sweet, good (N.A.)	1.549 ^c^	1.507 ^c^	0.690 ^c^	0.922 ^c^	2.375 ^b,c^	0.794 ^c^	1.197 ^c^	3.999 ^a,b^	4.506 ^a^	0.917 ^c^
			±0.359	±0.171	±0.288	±0.486	±0.049	±0.683	±0.033	±1.386	±2.405	±0.092
	Acids											
A1	Acetic acid **	Vinegar, floral, pungent, herbaceous (200) ^δ^	9.210 ^a,b^	8.460 ^a,b^	8.787 ^a,b^	5.531 ^b^	6.438 ^a,b^	3.734 ^b^	4.962 ^b^	4.353 ^b^	12.479 ^a^	3.342 ^b^
			±1.994	±2.156	±2.721	±0.012	±0.201	±1.547	±0.072	±1.206	±0.103	±1.029
		*Acids*										
A2	Propanoic acid **	Vinegar, flowery, soap (8.1) ^η^	0.008 ^a,b^	0.012 ^a^	0.010 ^a^	0.008 ^a,b^	0.012 ^a^	0.010 ^a,b^	0.009 ^a,b^	nd	0.013 ^a^	0.006 ^a,b^
			±0.002	±0.003	±0.001	±0.002	±0.003	±0.005	±0.001		±0.002	±0.001
A3	Isobutyric acid ***	Sweet, fruity, fresh (200) ^δ^	0.872 ^b,c^	1.387 ^a,b^	1.768 ^a^	1.022 ^a,b,c^	1.135 ^a,b,c^	1.098 ^a,b,c^	0.427 ^c^	0.485 ^c^	1.649 ^a,b^	0.790 ^b,c^
			±0.243	±0.166	±0.023	±0.021	±0.309	±0.329	±0.025	±0.237	±0.159	±0.098
A4	Butanoic acid **	Oily, not good (0.23) ^η^	0.218 ^b^	0.301 ^a,b^	0.490 ^a,b^	0.275 ^a,b^	0.601 ^a^	0.467 ^a,b^	0.279 ^a,b^	0.319 ^a,b^	0.486 ^a,b^	0.307 ^a,b^
			±0.033	±0.077	±0.139	±0.005	±0.044	±0.179	±0.006	±0.006	±0.016	±0.003
A5	Isovaleric acid ***	Toast, pastry, butter, cheese (0.0334) ^η^	2.861 ^a,b^	3.312 ^a^	3.128 ^a,b^	2.361 ^a,b,c^	1.037 ^c^	3.260 ^a^	3.287 ^a^	1.592 ^b,c^	1.969 ^a,b,c^	1.836 ^a,b,c^
			±0.697	±0.717	±0.323	±0.017	±0.238	±0.298	±0.043	±0.007	±0.012	±0.320
A6	Octanoic acid	Toasted bread (0.5) ^δ^	1.770	1.320	1.808	1.432	2.114	2.012	1.869	3.007	1.847	1.744
			±0.856	±0.217	±0.491	±0.061	±0.667	±0.029	±0.034	±0.237	±0.134	±0.280
		Aldehydes										
AD1	Hexanal^β^	Herbaceous, grass (0.0045) ^ϕ^	Nd	nd	nd	0.008	0.018	0.024	0.009	0.028	0.047	0.022
						±0.001	±0.001	±0.011	±0.001	±0.005	±0.009	±0.013
AD2	(Z)-3-Hexenal ^β^ **	Grass, green (0.00025) ^π^	0.033 ^a,b^	0.010 ^a,b^	0.014 ^a,b^	0.007 ^a,b^	0.015 ^a,b^	0.012 ^a,b^	nd	nd	0.043 ^a^	0.015 ^a,b^
			±0.011	±0.001	±0.003	±0.001	±0.002	±0.008			±0.021	±0.006
AD3	Methional ***	Fruity, sour, boiled vegetables (0.0005) ^ξ^	0.092 ^c^	0.134 ^b,c^	0.195 ^a,b^	0.126 ^b,c^	0.217 ^a^	0.152 ^a,b,c^	0.137 ^b,c^	0.187 ^a,b^	0.137 ^b,c^	0.095 ^c^
			±0.009	±0.031	±0.006	±0.001	±0.032	±0.016	±0.002	±0.014	±0.003	±0.013
		Dioxanes										
D1	2,5-Dimethyl-1,4-dioxane ^β^ **	Green, grass (2.5) ^σ^	0.094 ^a,b,c^	0.027 ^c^	0.043 ^c^	0.049 ^b,c^	0.060 ^a,b,c^	0.094 ^a,b,c^	0.046 ^c^	0.151 ^a^	0.104 ^a,b,c^	0.143 ^a,b^
			±0.053	±0.002	±0.002	±0.007	±0.006	±0.022	±0.006	±0.012	±0.026	±0.014
		Esters										
E1	Ethyl propanoate *	Fruity, toasted, sweet (0.55) ^ϕ^	1.398 ^c,d^	1.317 ^c,d^	1.469 ^b,c,d^	1.311 ^c,d^	1.783 ^a,b,c^	1.411 ^b,c,d^	0.998 ^d^	2.293 ^a^	2.142 ^a,b^	1.161
			±0.268	±0.353	±0.396	±0.044	±0.186	±0.732	±0.098	±0.398	±0.113	±0.024 ^c,d^
E2	Ethyl isobutyrate	Fruity, strawberry, lactic (0.015) ^ϕ^	1.438	1.289	1.275	1.118	1.123	1.054	1.034	1.423	1.634	0.874
			±0.257	±0.252	±0.225	±0.142	±0.028	±0.434	±0.062	±0.136	±0.012	±0.044
E3	Isobutyl acetate **	Alcohol, oily, fruity (1.6) ^μ^	0.125 ^b,c^	0.107 ^c^	0.169 ^a,b,c^	0.111 ^b,c^	0.210 ^a,b,c^	0.366 ^a^	0.274 ^a,b,c^	0.311 ^a,b^	0.270 ^a,b,c^	0.262 ^a,b,c^
			±0.070	±0.030	±0.033	±0.005	±0.025	±0.097	±0.014	±0.034	±0.021	±0.031
E4	Ethyl butyrate ***	Fruity, strawberry, soil (0.02) ^γ^	0.278 ^a^	0.039 ^b,c^	0.042 ^b,c^	0.082 ^b,c^	0.151 ^b^	0.105 ^b,c^	0.020 ^c^	0.042 ^b,c^	nd	0.057 ^b,c^
			±0.061	±0.014	±0.027	±0.035	±0.012	±0.031	±0.009	±0.001		±0.007
E5	Ethyl 2-methylbutyrate ***	Fruity, strawberry, sweet (0.018) ^γ^	0.057 ^b,c^	0.048 ^b,c^	0.042 ^c^	0.054 ^b,c^	0.034 ^c^	0.040 ^c^	0.075 ^a,b^	0.053 ^b,c^	0.098 ^a^	0.035 ^c^
			±0.011	±0.007	±0.004	±0.001	±0.003	±0.005	±0.001	±0.001	±0.007	±0.009
E6	Ethyl isovalerate **	Fruity, rubbish, jam (0.003) ^γ^	0.111 ^a^	0.073 ^a,b^	0.094 ^a,b^	0.088 ^a,b^	0.036 ^b^	0.086 ^a,b^	0.124 ^a^	0.137 ^a^	0.129 ^a^	0.071 ^a,b^
			±0.017	±0.012	±0.033	±0.003	±0.004	±0.013	±0.001	±0.014	±0.018	±0.019
E7	Isoamyl acetate ***	Banana, solvent (0.03) ^γ^	1.542 ^b,c,d^	1.068 ^d^	1.731 ^b,c,d^	1.509 ^c,d^	2.529 ^b^	4.768 ^a^	2.366 ^b,c^	4.687 ^a^	2.435 ^b,c^	4.385 ^a^
			±0.179	±0.223	±0.018	±0.030	±0.459	±0.393	±0.015	±0.218	±0.020	±0.005
E8	Ethyl 3-methylpentanoate ^β^	Cooked fruit, apple (0.08) ^χ^	Nd	nd	nd	nd	0.010	0.007	0.003	0.009	0.015	nd
							±0.002	±0.002	±0.001	±0.003	±0.003	
E9	Ethyl hexanoate **	Butter, cocoa powder, fruity (0.005) ^γ^	0.555 ^a,b,c^	0.455 ^c^	0.575 ^a,b,c^	0.526 ^a,b,c^	0.697 ^a,b,c^	0.726 ^a,b^	0.547 ^a,b,c^	0.758 ^a^	0.578 ^a,b,c^	0.496 ^b,c^
			±0.052	±0.083	±0.003	±0.007	±0.136	±0.034	±0.002	±0.011	±0.010	±0.029
E10	Ethyl octanoate **	Floral, sweet, charred wood (0.002) ^γ^	0.465 ^a,b^	0.285 ^b^	0.495 ^a,b^	0.384 ^a,b^	0.521 ^a,b^	0.595 ^a,b^	0.390 ^a,b^	0.678 ^a^	0.425 ^a,b^	0.429 ^a,b^
			±0.139	±0.050	±0.052	±0.001	±0.116	±0.040	±0.001	±0.025	±0.001	±0.089
E11	Ethyl decanoate **	Floral, sweet, iodine (0.2) ^γ^	0.032 ^c^	0.052 ^b^	0.041 ^b,c^	0.080 ^c^	0.052 ^b,c^	0.042 ^c^	0.043 ^c^	0.048 ^c^	0.163 ^a^	0.037 ^c^
			±0.002	±0.008	±0.009	±0.003	±0.003	±0.014	±0.001	±0.007	±0.006	±0.005
E12	Ethyl 4-hydroxybutanoate ***	Alcohol, fruity (40) ^Ψ^	3.618 ^c^	4.163 bc	7.304 ^a,b,c^	5.305 ^b,c^	10.762 ^a^	7.429 ^a,b,c^	3.033 ^c^	5.172 ^b,c^	8.803 ^a,b^	5.307 ^b,c^
			±0.978	±0.731	±0.539	±0.224	±1.409	±2.710	±0.008	±1.233	±0.044	±0.676
E13	Phenethyl acetate ***	Wood, spiced, floral (0.25) ^η^	0.238 ^c^	0.163 ^c^	0.188 ^c^	0.202 ^c^	0.330 ^b,c^	0.465 ^b^	0.338 ^b,c^	0.718 ^a^	0.326 ^b,c^	0.506 ^b^
			±0.059	±0.029	±0.005	±0.004	±0.064	±0.019	±0.017	±0.008	±0.010	±0.091
E14	Ethyl dodecanoate	Sweet, peppery, foot smell (0.5) ^ϕ^	nd	nd	0.020	0.016	nd	0.033	0.021	0.030	nd	0.026
					±0.002	±0.001		±0.008	±0.001	±0.005		±0.001
E15	Ethyl hydrogen succinate **	Fruity, moss, charred wood (1000) ^φ^	36.990 ^a,b^	50.923 ^a^	42.683 ^a,b^	31.943 ^a,b^	19.520 ^b^	33.334 ^a,b^	23.676 ^a,b^	21.518 ^a,b^	37.467 ^a,b^	19.986 ^b^
			±11.046	±9.238	±8.604	±0.463	±1.726	±11.666	±0.570	±4.323	±0.116	±3.594
		Furans										
F1	Furfural **	Pastry, toast (14.1) ^γ^	0.228 ^a^	0.144 ^a,b^	0.142 ^a,b^	0.191 ^a,b^	0.107 ^a,b^	0.065 ^b^	0.082 ^a,b^	0.106 ^a,b^	0.227 ^a^	0.078 ^a,b^
			±0.089	±0.010	±0.040	±0.014	±0.023	±0.019	±0.013	±0.019	±0.038	±0.004
		Ketones										
K1	2,3-Butanedione *	Toffee, caramel, lactic, butter (0.1) ^η^	0.767 ^c^	0.528 ^c^	0.797 ^b,c^	0.672 ^c^	1.542 ^a^	1.781 ^a^	0.659 ^c^	1.142 ^a,b,c^	1.085 ^a,b,c^	1.489 ^a,b^
			±0.310	±0.133	±0.031	±0.084	±0.380	±0.777	±0.091	±0.332	±0.150	±0.047
K2	2,3-Pentanedione***	Caramel, sweet, butter, brown sugar (0.03) ^Ω^	0.089 ^c,d^	0.040 ^d^	0.100 ^c,d^	0.208 ^b,c^	0.355 ^b^	0.567 ^a^	0.189 ^c,d^	0.220 ^b,c^	0.174 ^c,d^	0.344 ^b^
			±0.080	±0.016	±0.020	±0.007	±0.105	±0.142	±0.008	±0.045	±0.029	±0.067
		Phenols										
Ph1	Eugenol **	Clove, spiced, floral (0.006) ^γ^	0.265 ^c,d,e^	0.549 ^a^	0.296 ^c,d,e^	0.308 ^b,c,d^	0.158 ^e^	0.250 d,e	0.442 ^a,b^	0.390 ^b,c^	0.253 ^c,d,e^	0.282 ^c,d,e^
			±0.120	±0.120	±0.047	±0.014	±0.046	±0.006	±0.008	±0.001	±0.021	±0.066
		Pyrazines										
Py1	2,3-Diethyl-5-methylpyrazine ^β^	Vinegar, meaty, toast (0.018) ^ω^	0.031	nd	0.072	nd	0.084	0.024	0.010	nd	nd	0.017
			±0.003		±0.047		±0.004	±0.001	±0.001			±0.005
Py2	2-Ethyl-3,5-dimethylpyrazine ^β^ *	Herbaceous, acetic (0.0075) ^ω^	0.030 ^c,d^	0.080 ^a^	0.064 ^a,b^	0.029 ^c,d^	nd	0.029 ^c,d^	0.012 ^d^	0.047 ^a,b,c^	0.030 ^b,c,d^	0.021 ^c,d^
			±0.004	±0.029	±0.018	±0.002		±0.019	±0.001	±0.029	±0.003	±0.002
		Unknown										
Un1		Vinegar, grass, oily	0.010	nd	nd	0.037	0.060	0.031	0.025	0.081	0.089	0.048
			±0.008			±0.001	±0.001	±0.013	±0.003	±0.028	±0.009	±0.001
Un2		Metallic, not good, foot smell	0.039	nd	nd	0.031	nd	nd	0.018	0.072	0.085	0.047
			±0.032			±0.006			±0.001	±0.022	±0.013	±0.008
Un3	**	Smoke, burning	0.026 ^b,c^	nd	nd	0.022 ^b,c^	0.024 ^b,c^	0.019 ^b,c^	0.016 ^c^	0.042 ^a^	0.032 ^a,b^	0.022 ^b,c^
			±0.007			±0.002	±0.004	±0.008	±0.002	±0.014	±0.011	±0.002
Un4	*	Floral, herbal, sweet	0.074 ^d^	0.117 ^a,b,c^	0.128 ^a,b^	0.091 ^b,c,d^	0.090 ^c,d^	0.092 ^b,c,d^	0.098 ^b,c,d^	0.146 ^a^	0.150 ^a^	0.094 ^b,c,d^
			±0.027	±0.033	±0.007	±0.001	±0.014	±0.022	±0.014	±0.003	±0.006	±0.009
Un5		Spiced, soap, chocolate	Nd	nd	nd	nd	0.068	0.213	nd	0.130	0.254	0.121
							±0.002	±0.092		±0.006	±0.012	±0.001
Un6		Wet stone, fermenting fruit	Nd	nd	0.812	0.554	0.718	0.621	0.305	0.533	0.483	0.278
					±0.416	±0.026	±0.063	±0.265	±0.007	±0.094	±0.002	±0.030

^α^ Identification letters; ^β^ Compound not confirmed; ^δ^ [25]; ^γ^ [20]; ^η^ [26]; ^φ^ [27]; ^ϕ^ [28]; ^λ^ [29]; ^μ^ [30]; ^ρ^ [31]; ^π^ [32]; ^ν^ [33]; ^σ^ [34]; ^χ^ [35]; ^Ψ^ [36]; ^Ω^ [37]; ^ω^ [38]; ^ξ^ [39]; Un—Unknown; NA—Not available; nd—Not detected; * significant; ** very significant; *** highly significant; ^a,b,c,d,e^-classes determined by the post-hoc differential test of Bonferroni at a *p* < 0.05.

### 2.4. Odor Active Compounds

Odor threshold values and odor activity values (OAVs) are frequently used to show the relative contribution and the relative importance of the identified and quantified volatile odor compounds in the wines’ aromas [40]. To determine the effective odor active compounds (OACs) in the analyzed monovarietal red wines, and build, for each variety, an aroma characterization, OAVs were determined as the quotient between the concentration of each identified compound in each variety and its odor perception threshold. Of the VOCs identified, 24 have been established as OACs and grouped in Table 4, which shows the OACs, chemical species, identification code, most used aromatic descriptors during the GC-O analysis, odor series assigned, and name of the OACs identified. Even if the compounds with an OAV ≤ 1 can have a synergic, additive, or antagonistic effects on the aroma profile of wines [5], only the compounds with an OAV ≥ 1 have been considered as OACs. 

To investigate the correlation between the sample and the OACs, and to better understand the similarities/differences among the samples, a PCA was conducted, considering as observations the monovarietal red wines samples, and as a variable the odor activity values of the compounds listed in Table 4. 

**Figure 6:**
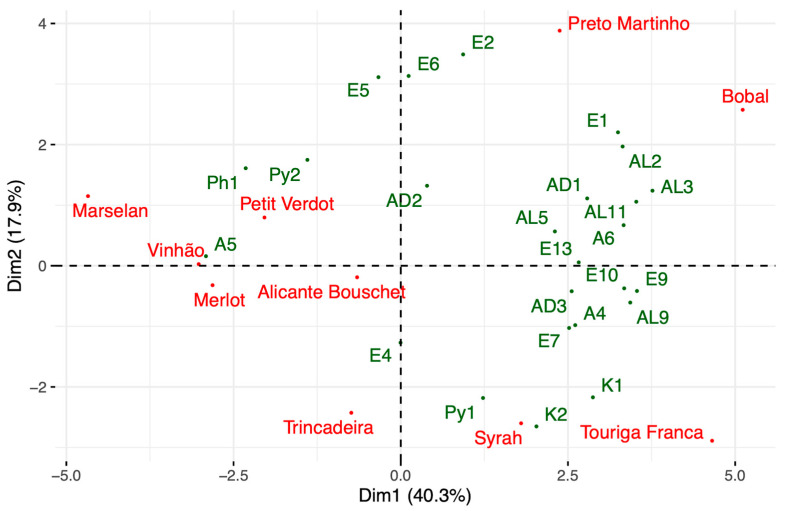
Projection of the wine samples and OACs in the plane defined by two components of the standardized PCA; identification of the OACs in Table 3.

As can be seen in Figure 6, the first two dimensions show a cumulative variance of 58.2% with 40.3% from Component 1 and 17.9% from Component 2. The greatest significance for Component 1 is assumed by the variables isoamyl alcohols, ethyl hexanoate, 2-phenyletanol, 3-mercaptohexanol, ethyl octanoate, octanoic acid, isobutanol, ethyl propanoate, 2,3-butanedione, hexanal, phenylethyl acetate, butanoic acid, and isovaleric acid. Ethyl isobutyrate, ethyl isovalerate, ethyl 2-methylbutyrate and 2,3-pentanedione showed the greatest importance for Component 2.

By analyzing the result of the PCA depicted in Figure 6, it can be observed that the distribution pattern of the wine samples between the components is not too dissimilar to that already observed in the multivariate analyses carried out previously. Furthermore, as can be seen from the visual comparison of the multivariate analyses, these reflect, with exceptions, the grouping of the samples based on the ANOVA performed on the attribute ‘General Appreciation’ evaluated during the sensory analysis. This might suggest that in the samples analyzed, although they are red wines, the volatile component, or at least some elements related to it, are significantly correlated with the tasting panel’s overall appreciation of the product [41]. This element constitutes a starting point for future studies since, although the VOCs and more specifically the OACs were influenced by the climatic identification of the place where the grapes were grown (leading to a relative flattening of the odor profile of wines characterized mainly by floral, fruity, and vegetal/oleic scents), an influence of olfactory volatile compounds on the determination of the appreciation of the products analyzed seems nevertheless evident. 

### 2.5. Aromatic Characterization

After the computation of the several collected data, a systematic aromatic characterization of the monovarietal red wines, produced with a cultivar grown in a hot climate territory and selected for their adaptation to abiotic stress, have been proposed. The samples have been divided into three groups based on the similarity of the odorous profile. 

The first class includes all wine samples that showed a more complex odor profile during the various analyses conducted. In this group are the wines produced with the cultivars ‘Vinhão’, ‘Merlot’, ‘Marselan’ and ‘Alicante Bouschet’. These wines showed the greatest appreciation by the judges during the sensory analysis, also revealing the highest scores for the attribute ‘Intensity of Odor’. Concerning the ‘Merlot’ variety, these results agree with high plasticity in response to climate change, reported by other researchers [40] to this variety. Looking at the data from the general chemical analysis, these samples also showed similar patterns in terms of alcoholic strength, titratable acidity and reducing substances. Concerning the concentration of reducing substances (the highest found in the sample analyzed), it must be said that it seems to be a figure in contrast to the high appreciation of the wines achieved in the sensory analysis, but this could be explained by taking into account that the samples also show to be rich in compounds that are responsible for the perception of body in the mouth, a sensation that could have masked the high concentration of residual sugars [42]. This may open possibilities for future studies in which the harvest process may be anticipated, achieving sugar levels not as high as in this case. 

The ‘Petit Verdot, ‘Syrah‘ and ‘Trincadeira‘ wines were grouped in the second class, showing, during the sensory and chemical analysis performed in this study, similar characteristics and appearing to have a simpler sweet/fruity bouquet (compared to the wines enrolled in the first class).

In the last class, the wines that obtained the lowest scores in the attribute ‘General Appreciation’ during the SA have been grouped and were characterized by a greener/pungent aroma profile. The wines obtained from the cultivars ‘Touriga Franca’, ‘Preto Martinho’ and ‘Bobal’ belong to this class.

Regarding the OACs, each monovarietal wine presents a similar profile in terms of molecules identified (being methional, 3-mercaptohexanol, ethyl octanoate, ethyl hexanoate, isovaleric acid, ethyl isobutyrate, eugenol, and isoamyl acetate—the VOCs with higher odor activity values). The differences among the groups and the wines are related to the relative odor activity values differing from one variety to another, and to the presence of fewer impact VOCs that act as a fine regulator in the profile of the wines [5]. 

As far as is known by the authors, the wines produced from the cultivars ‘Alicante Bouschet’ and ‘Preto Martinho’ have been aromatically characterized for the first time in this study, while for the other cultivars there exist in the literature works that show similarities and differences with the characterization proposed, e.g., for the cultivar ‘Vinhão’ [43], for the cultivar ‘Merlot’ [25], for the cultivar ‘Marselan’ [44], for the cultivar ‘Petit Verdot’ [20], for the cultivar ‘Syrah’ [45], for the cultivar ‘Trincadeira’ [46,47], for the cultivar ‘Touriga Franca’ [48] and for the cultivar ‘Bobal’ [18]. The enlightened differences could be mainly attributed to ‘terroir’ factors, the climatic trend of the producing vintage, and the different analytical approaches used in the different studies.

This research was a first approach to determine the volatile compounds characterizing the varieties in question, and for some varieties, this is the first systematic characterization of the aroma compounds ever conducted in Portugal. However, in spite of interesting results, the major drawback of this study comes from the fact that the results are obtained from only one harvest, since several studies have verified the influence of vintage in the wines’ volatile composition [23,47]. Further studies are necessary to better understand the interaction between the aromatic profile of the wines and their appreciation to provide a clearer picture for producers and consumers. Moreover, following the behavior of the varieties characterized in this work through the years (under different weather conditions, harvesting the grapes at different and more homogenous ripening levels, evaluating the use of dissimilar yeast strains, and applying different analytical techniques and strategies) is a subject that remains unstudied.

## 3. Materials and Methods

### 3.1. Vineyard and Wine Production

The ‘Petit Verdot’, ‘Marselan’, ‘Merlot’, ‘Touriga Franca’, ‘Syrah’, ‘Vinhão’, ‘Bobal’, ‘Preto Martinho’, ‘Trincadeira’, ‘Alicante Bouschet’, and grapes used for the vinification were obtained from the Ampelographic field of Esporão located in Herdade do Esporão, Reguengos de Monsaraz, Distrito de Évora, Alentejo, Portugal, one of the hottest Portuguese wine regions. The 9-year-old vineyard, grafted on 1103 P, was planted in a Eutric Cambisol with an ApBw1Bw2C profile, derived from granite, with 75–80% of sand. The training system used was vertical shoot positioned system. Row orientation was North-South with 3 m between rows and 1.5 m between vines. Irrigation (surface drip irrigation with one dripper per meter at 2.4 L/h) was done weekly and the irrigation amount (around 100 mm from pea size to maturation) was determined using the FAO-56 approach [49], with crop coefficients derived from a vegetation index (NDVI) and imposing a stress coefficient of 0.5.

During the 2020 growing cycle, the average temperature, rainfall and reference evapotranspiration were 20.5 °C, 356.4 mm and 985.8 mm, respectively. During the maturation period, the average maximum and minimum temperature were 36.2 and 17.4 °C, respectively. For each variety, around 60 kg of grapes was harvested, transported to the experimental winery of INIAV (Dois Portos), kept at 4 °C and processed one day after the harvest. For each variety, the harvested bunches, after being weighed, were destemmed and crushed. The total volume of the monovarietal must was divided into two similar batches and convoyed in two stainless steel microvinifiers of about 40 to 60 L of total volume, with the addition of 10 mg/L of a solution of 70% potassium metabisulfite and 30% L-ascorbic acid (Oxyless produced by Perdomini-IOC S.p.A., Varese, Italy). The must was successively inoculated with a concentration of 0.3 g/L of selected active dry yeast *Saccaromyces cerevisiae* (uvaferm bdx™ produced by Lallemand Inc., Montréal, QC, Canada) at a controlled temperature of 25 °C. One day after the inoculation of the yeast, the must was sequentially inoculated with selected malolactic bacteria of the species *Oenococcus oeni* (lalvin vp41^®^ produced by Lallemand Inc., Montréal, Canada) during a pumping over without aeration. After the descent of 30 points of density, the fermenting must was supplemented with 0.3 g/L of a nutrient mixture (fermaid e™ produced by Lallemand Inc., Montréal, Canada and composed by inactivated yeast *Saccharomyces cerevisiae,* mineral salts, and vitamins) during the execution of a delestage. Once we completed the alcoholic fermentation process, the wines were racked and the pomace was pressed. One day after the first rack, the wines were racked for a second time and transferred into 20 L demijohns. After the end of the malolactic fermentation (MLF), each wine was combined with 150 mg/L of a commercial mixture of sulphur dioxide and ascorbic acid (Oxyless from Perdomini-IOC S.p.A.), and the wines were bottled without filtration during the month of March 2021. 

A total of 20 wines were produced, and after the bottling, these wines were sampled to proceed with the sensory and physic-chemical analyses.

### 3.2. Wine General Analysis

For each sample, according to the official analytical methods [50], the following analytical determinations were performed: density (ρ20), alcoholic strength, total acidity (TA), volatile acidity (VA), fixed acidity (FA), reducing substances, sulphur dioxide and pH.

### 3.3. Sensory Analysis

The sensory evaluation of the samples was performed in the sensory analysis laboratory of INIAV (Dois Portos, Portugal) using the ‘Attribute Rating’ sensory method [51]. The panel was composed of nine trained members—three females and six males—aged from 29 to 57, who were members of the INIAV research group. Every panelist analyzed the samples in an individual workstation equipped with a light, a sink, and white surfaces (ISO 8589:2007). The wine samples were analyzed using ISO tulip glasses (ISO 3591:1977) in which a volume of about 50 mL of each sample was poured. 

The evaluation was carried out using a structured discontinue scale and the choice of the attribute to be rated was based on the works of Botelho [46]. The evaluated attributes included visual, olfactory, and attributes (rated with a structured scale that goes from 0 to 10, where 0 is associated with the absence of the attribute while 10 is associated with the maximum intensity of the attribute), and general appreciation (rated with a structured scale that goes from 0 to 20 where 0 is associated with no quality of the sample while 20 is associated with maximum quality of the sample, where for quality is intended the perception of the wine in terms of balance and absence of defects). The color attributes rated were: ‘Limpidity’, ‘Color Intensity/Brilliance’, and ‘Color Quality’. The odor attributes rated were: ‘Red Fruits/Berries’, ‘Dried Fruits’, ‘Cooked Fruits/Jam’, ‘Vegetal/Herbaceous’, ‘Spiced’, ‘Chocolate’, ‘Smoke/Toasted’, ‘Floral’, and ‘Odor Intensity’. The gustatory attributes rated were: ‘Acidity’, ‘Sweetness’, ‘Bitterness’, ‘Astringency’, ‘Body’, ‘Complexity’, ‘Length/Finish’ (flavor persistency).

The samples, identified with a three-digit code, were presented anonymously following a balance order with the aim to eliminate the first-order carryover effects [52].

Given the pandemic situation, each panelist went in the tasting room with a mask and only removed it when they were already seated and started the sensory test. In order to respect the rules of the health authorities, the tasting room was used only at 50% of its capacity in order to ensure the distance among panelists.

### 3.4. Volatile Odorous Compounds Analysis

#### 3.4.1. Reagents

Anhydrous sodium sulfate was purchased from AppliChem GmbH (Darmstad, Germany); dichloromethane (99.9%) was supplied by Honeywell Riedel-de Haën (Steinheim, Germany) and silanized glass wool by Supelco (Steinheim, Germany). 2-Octanol (≥99.5%), used as an internal standard was purchased from Sigma-Aldrich (St. Louis, MO, USA).

GC-FID and GC-MS used standards: acetic acid was purchased from Riedel-de-Haen (Seelze, Germany); isobutanol, 2-methyl-1-butanol, 3-methyl-1-butanol,benzyl alcohol, 2-phenylethanol,propanoic acid, butanoic acid, isovaleric acid, octanoic acid, hexanal, ethyl propanoate, isobutyl acetate, ethyl 2-methylbutyrate, ethyl hexanoate, ethyl decanoate, phenethyl acetate, ethyl dodecanoate, eugenol were acquired from Fluka (Buchs, Switzerland);1-propanol, 2,3-butanediol, 1,2-propanediol, glycerol, isobutyric acid, ethyl butyrate, ethyl octanoate were purchased from Merck (Darmstadt, Germany); e-ethoxy-1-propanol, methional, ethyl isobutyrate, ethyl isovalerate, isoamyl acetate, ethyl hydrogen succinate, furfural were bought from Aldrich (Steinheim, Germany) and 2,3 butanedione was acquired from Sigma-Aldrich (Saint Louis, MO, USA). 

#### 3.4.2. Gas Chromatography Olfactometry Analysis

##### Liquid–Liquid Extraction Procedure

The free volatile compounds were extracted from wine samples (50 mL) using discontinuous ultrasound liquid–liquid extraction with dichloromethane, dried over anhydrous sodium sulphate and then concentrated to 0.20 mL according to the described method [27], adapted from Cocito, et al. [53]. The wine extraction was performed in duplicate, and the extracts were stored at −20 °C until analysis by gas chromatography olfactometry (GC-O) and gas chromatography mass spectrometry (GC-MS). 

##### Gas-Chromatography Olfactometry Procedure

The GC-O system consisted of an Agilent Technologies 6890 Series chromatograph (Wilmington, DE, USA) equipped with a flame ionization detector (FID), an Olfactory Detection Port (ODP 2 Gerstel, Mülheim an der Ruhr, Germany), and an Olfactory Intensity Device (OID 1, Gerstel, Mülheim an der Ruhr, Germany). GC effluent was split 1:3 between the FID and the ODP. Each extract sample (0.4 μL) was injected (splitless) into a capillary column (DB-WAX, 30 m length × 0.320 mm i.d. × 0.50 μm, Agilent J&W Technologies, CA, USA). Operating conditions were as follows: injector, 250 °C with an inlet pressure of 9.1 psi; FID, 260 °C; ODP, 220 °C; carrier gas hydrogen (H_2_ ≥ 99.9992%), 2.4 mL min^−1^; oven temperature program: 35 °C for 6 min, 3.5 °C min^−1^ until 55 °C, 10 °C min^−1^ up to 85 °C, 7.5 °C min^−1^ until 100 °C, 10 °C min^−1^ up to 130 °C, held for 1 min, 5 °C min^−1^ up to 210 °C held for 30 min. The Kovats indices (KIs) of the compounds were calculated from the retention time of n- alkanes (C7–C26, C28 and C30) [54]. 

The gas-chromatographic analysis was conducted using the previously described conditions [22] based on the distribution of thresholds among judges (detection frequency) entrenched on the Nasal Impact Frequency strategy [5]. The panel of the sniffers was composed by nine trained persons—four males and five females, aged from 26 to 57, who were members of the INIAV research group. Considering the pandemic situation, the GC-O acquisition computer was moved to a contiguous room, separated from the GC-O equipment room by a glass, in order to ensure the separation between the GC operator and the sniffer. Between each sniffer session, the olfactometer button, the seat and the table were clean and disinfected. The detection frequency was used as an estimate of the odors’ intensity, and only when the detection frequency was superior to 3 was the detected odor considered as valid and the related compound considered as a probable odor active compound (pOAC) [55]. To better visualize the contribution of the compounds to the aromatic profile of each wine, every pOAC, based on the main aroma description, was grouped into one of the eleven selected odor series (‘Chemical’, ‘Earth’, ‘Fatty’, ‘Floral’, ‘Fruity’, ‘Herbaceous’, ‘Pastry’, ‘Pungent’, ‘Roasting’, ‘Spiced’, and ‘Sweet’). Based on the odor series, the sum of the detection frequency of the pOACs (∑ Frequencies) was used to show the odor series contribution on the aroma profile of the wine [56,57].

#### 3.4.3. Identification of the Probable Odor Active Compounds

The GC-MS equipment consisted of a Finnigan MAT Magnum (San Jose, California, USA). An aliquot of 0.2–0.4 μL was injected and volatile compounds were separated using a fused silica capillary column of polyethylene glycol (HP-INNOWAX, 30 m length × 0.25 mm i.d. × 0.25 μm film thickness, Agilent J&W Technologies, CA, USA). Operating conditions were as follows: injector and transfer line temperature, 250 °C; carrier gas helium (He ≥ 99.9992%) with an inlet pressure of 12 psi and split ratio 1:60; the oven worked at a temperature program of 3.5 °C min^−1^ of 35 °C (6 isothermal min), to 55 °C, 7.5 °C min^−1^ until 130 °C, 5 °C min^−1^ up to 210 °C and held at this temperature for 30 min. The mass spectrometer was equipped with an ion trap detector and was operated in the electron impact mode at 70 eV, scanning at full scan mode in the range *m*/*z* 40–340. 

The identification of the compounds was systematically confirmed by comparing the chromatogram obtained with the GC-MS data (confirmed comparing the mass spectra obtained with those of the libraries of NIST and Wiley), by confronting the KIs calculated with the indices contained in the NIST libraries or in the scientific literature [5,22,27,58], and, when possible, with the retention indices of pure standard compounds available, obtained under the same analytic conditions.

#### 3.4.4. Quantification of the Probable Odour Active Compounds

##### Liquid–Liquid Micro-Extraction Procedure

The extraction of the free volatile compounds from the wines to be analyzed through GC-FID technique was based on a Liquid–Liquid Micro-Extraction (LLME) following a previously adapted method [59]. The extracts were stored at −20 °C until analysis.

##### Gas Chromatography Flame Ionization Detector Procedure

The GC-FID system consisted of an Agilent Technologies 6890 N GC System (Santa Clara, CA, USA) equipped with a flame ionization detector (FID). Each sample (1.4 μL) was injected using splitless mode into a high polarity capillary column (HP-INNOWAX, 30 m length × 0.320 mm i.d. × 0.25 μm Polyethylene glycol (PEG) film thickness, Agilent J&W Technologies, Santa Clara, CA, USA). Operating conditions were as follows: injector, 250 °C, with an inlet pressure of 5.3988 psi; FID, 260 °C; carrier gas Hydrogen (H_2_ ≥ 99.9992%), 2.4 mL min^−1^; oven temperature program: 35 °C held for 6 min, increase at 3.5 °C min^−1^ to 55 °C; 7.5 °C min^−1^ until to 130 °C; 5 °C min^−1^ up to 210 °C, held for 30 min. The Kovats indices (KIs) of the compounds were calculated as explained previously [55].

The quantification of volatiles, as 2-octanol equivalents, was performed by comparing retention indexes with those of pure standard compounds when available and assuming a response factor of one between the internal standard (IS) and the analytes.

#### 3.4.5. Odor Activity Values

The odor activity value (OAV) of each probable odor active compound (pOAC) was calculated by the ratio between the perception threshold of the compound (found in the scientific literature) and its relative concentration in each sample. Only the compounds with an OAV ≥ 1 were considered as odor active compounds (OACs).

#### 3.4.6. Data and Statistical Analysis

One-way analysis of variance (ANOVA) was performed taking the wine variety as a fixed factor, followed by the post-hoc differential test of Bonferroni at a *p* < 0.05 for the mean comparison when a significant effect of the factor was detected. Principal component analysis (PCA) and hierarchical cluster analysis (HCA) were also applied to the obtained data (sensory and volatile results). The statistical analysis was conducted using ‘R: A language and environment for statistical computing’ (version 3.6.3—2020, R Foundation for Statistical Computing, Vienna, Austria) software. Excel (version 16.63.1—2022, Microsoft, Redmond, Washington, DC, USA) and R software were used to draw the figures and the tables.

## 4. Conclusions

This study was conducted with the objective of quantifying the oenological potential of the varieties more adapted to a Climate Change scenario cultivated in a dry and warm climate region (Alentejo, Portugal). Ten monovarietal wines, produced from varieties selected for their adaptability in a global warming scenario (‘Alicante Bouschet’, ‘Bobal’, ‘Marselan’, ‘Merlot’, ‘Petit Verdot’, ‘Preto Martinho’, ‘Syrah’, ‘Touriga Franca’, ‘Trincadeira’, and ‘Vinhão’), in terms of water use efficiency, vigor, tolerance to heatwaves, and and yield, among other things, were characterized according to their aroma through both sensory analysis and several gas chromatography approaches. Based on the interpolation of the results of the various statistical analyses carried out, three classes of similarity/difference between the sensory profiles were identified: ‘Complex Bouquet’ wines, ‘Simple Fruity’ wines, and ‘Green/Pungent’ wines. Forty-nine probable odor active compounds were identified and based on the odor activity values, of them, twenty-four were recognized as odor active compounds, classified mainly as VOCs of fermentation origin. An aromatic characterization of the monovarietal wines has been proposed identifying a similar profile in terms of odor active compounds, with methional, 3-mercaptohexanol, ethyl octanoate, ethyl hexanoate, isovaleric acid, ethyl isobutyrate, eugenol, and isoamyl acetate being the principal volatile odor compounds.

## Figures and Tables

**Table 1:** Results of the generic chemical analysis of the monovarietal red wines. [‘Petit Verdot’ (PV), ‘Touriga Franca’ (TF), ‘Preto Martinho’ (PM), ‘Bobal’ (B), ‘Marselan’ (MA), ‘Alicante Bouschet’ (AB), ‘Syrah’ (SY), ‘Trincadeira’ (TR), ‘Merlot’ (ME), ‘Vinhão’ (VI)].

Monovarietal Red Wines										
Analytical Determination	PV	MA	AB	ME	SY	TF	VI	B	PM	TR
Density (ρ20) (g/cm^3^) ***	0.9926 ^b,c,d^ ±0.0001	0.9933 ^b,c^ ±0.0013	0.9930 ^b,c,d^ ±0.0008	0.9922 ^b,c,d^ ±0.0002	0.9911 ^c,d^ ±0.0001	0.9939 ^a,b^ ±0.0001	0.9956 ^a^ ±0.0001	0.9936 ^a,b^ ±0.0001	0.9927 ^b,c,d^ ±0.0001	0.9911 ^d^ ±0.0001
Alcoholic strength (% vol) ***	14.3 ^d^ ±0.1	16.1 ^a,b^ ±0.6	16.9 ^a^ ±0.1	16.2 ^a,b^ ±0.7	15.8 ^a,b,c^ ±0.1	14.7 ^c,d^ ±0.1	14.8 ^c,d^ ±0.1	11.6 ^e^ ±0.1	14.4 ^d^ ±0.1	15.0 ^b,c,d^ ±0.1
Total Acidity (g Tartaric acid/L) ***	6.57 ^a^ ±0.02	5.68 ^c^ ±0.05	5.37 ^d^ ±0.15	5.28 ^d^ ±0.01	4.93 ^e^ ±0.01	4.68 ^e^ ±0.02	6.20 ^b^ ±0.11	4.83 ^e^ ±0.06	5.52 ^c,d^ ±0.04	4.24 ^f^ ±0.01
Volatile Acidity (g Acetic acid/L) ***	0.99 ^a^ ±0.06	0.68 ^b^ ±0.01	0.70 ^b^ ±0.01	0.72 ^b^ ±0.01	0.43 ^c^ ±0.01	0.69 ^b^ ±0.01	1.00 ^a^ ±0.02	0.40 ^c^ ±0.01	0.90 ^a^ ±0.01	0.42 ^c^ ±0.01
Fixed Acidity (g Tartaric acid/L) ***	5.33 ^a^ ±0.06	4.84 ^b^ ±0.04	4.50 ^c^ ±0.16	4.39 ^c^ ±0.01	4.40 ^c^ ±0.01	3.82 ^d^ ±0.02	4.97 ^b^ ±0.09	4.33 ^c^ ±0.05	4.40 ^c^ ±0.04	3.71 ^d^ ±0.01
Reducing substances (g/L) ***	4.61 ^c,d,e^ ±0.93	10.03 ^a,b^ ±1.94	9.40 ^a,b^ ±1.68	8.24 ^b,c^ ±0.95	3.53 ^d,e^ ±0.01	3.35 ^d,e^ ±0.05	12.85 ^a^ ±0.07	2.22 ^e^ ±0.01	6.65 ^b,c,d^ ±0.08	3.75 ^d,e^ ±0.11
Free sulfur dioxide (mg/L)	15 ±2	14 ±1	12 ±1	15 ±1	20 ±5	23 ±6	17 ±3	19 ±5	18 ±1	17 ±1
pH ***	3.74 ^f^ ±0.04	3.65 ^g^ ±0.01	3.94 ^b,c^ ±0.01	3.87 ^c,d^ ±0.01	3.96 ^b^ ±0.02	4.28 ^a^ ±0.03	3.74 ^f^ ±0.02	3.77 ^e,f^ ±0.01	3.84 ^d,e^ ±0.01	3.93 ^b,c^ ±0.01

*** Highly significant (*p* < 0.001); ^a,b,c,d,e,f,g^—classes determined by the post-hoc differential test of Bonferroni at a *p* < 0.05.

**Table 2:** ANOVA of sensory results and the corresponding radar representation of the sensorial profile of the monovarietal red wines produced from the cv. ‘Petit Verdot’ (PV), ‘Touriga Franca’ (TF), ‘Preto Martinho’ (PM), ‘Bobal’ (B), ‘Marselan’ (MA), ‘Alicante Bouschet’ (AB), ‘Syrah’ (SY), ‘Trincadeira’ (TR) ‘Merlot’ (ME), and ‘Vinhão’ (VI).

Sensory Attribute	Monovarietal Red Wines									
	PV	MA	AB	ME	SY	TF	VI	B	PM	TR
Limpidity	8.8	8.5	9.0	8.2	9.0	9.1	8.8	9.4	9.2	9.2
Color intensity	8.9	9.4	9.2	9.2	9.2	9.0	9.4	7.9	6.8	8.1
Color quality	9.1	9.2	9.4	9.0	9.3	9.2	9.4	8.3	7.0	8.8
Red fruit/Berries	4.2	5.0	4.9	5.1	3.8	4.2	4.7	3.6	3.0	3.9
Dried fruits	1.3	2.1	1.7	1.7	1.5	1.2	1.7	1.2	1.5	1.7
Cooked fruits/Jam *	3.5 ^a,b^	3.4 ^a,b^	3.5 ^a,b^	4.0 ^a^	2.4 ^b,c^	3.2 ^a,b,c^	2.9 ^a,b,c^	2.5 ^b,c^	2.1 ^c^	2.1 ^c^
Vegetal/Herbaceous	0.7	1.0	1.0	1.0	1.4	1.8	1.6	2.1	1.9	2.2
Spiced	1.4	1.8	1.2	2.2	1.6	1.1	1.5	1.3	1.7	1.3
Chocolate	1.1	1.2	1.3	1.2	1.0	0.9	0.8	0.4	0.4	0.6
Smoke/Toasted	0.3	0.5	1.2	1.2	1.0	0.7	0.5	0.6	1.3	0.9
Floral	0.5	1.1	1.1	0.4	0.9	0.9	1.0	1.3	1.0	1.1
Odor Intensity	6.2	6.9	6.3	7.4	5.8	6.0	7.3	5.7	5.5	5.6
Acidity **	4.7 ^a^	2.9 ^c^	3.7 ^a,b,c^	3.0 ^b,c^	4.5 ^a,b^	3.8 ^a,b,c^	3.7 ^a,b,c^	3.8 ^a,b,c^	3.9 ^a,b,c^	3.5 ^a,b,c^
Sweetness **	3.2 ^a,b^	4.4 ^a^	3.5 ^a,b^	3.7 ^a,b^	2.5 ^a,b^	2.3 ^b^	3.6 ^a,b^	2.1 ^b^	2.2 ^b^	2.4 ^a,b^
Bitterness	3.1	2.8	3.4	2.4	3.3	3.4	2.9	3.1	3.2	3.6
Astringency	5.4	4.3	5.3	3.9	4.6	5.5	5.1	4.5	4.3	4.0
Body ***	5.5 ^a,b,c^	6.2 ^a,b^	6.3 ^a^	6.3 ^a^	5.8 ^a,b,c^	6.1 ^a,b^	6.2 ^a,b^	5.2 ^b,c^	4.8 ^c^	5.5 ^a,b,c^
Complexity **	5.5 ^c,d^	6.9 ^a^	6.5 ^a,b^	6.8 ^a^	5.7 ^b,c,d^	6.1 ^a,b,c^	7.0 ^a^	5.0 ^d^	5.2 ^c,d^	5.5 ^c,d^
Length/Finish **	5.8 ^c^	7.1 ^a^	6.8 ^a,b^	7.1 ^a^	6.0 ^b,c^	6.1 ^b,c^	7.1 ^a^	5.5 ^c^	5.6 ^c^	5.9 ^b,c^
General Appreciation ***	13.0 ^b,c,d^	14.8 ^a,b,c^	14.5 ^a,b,c^	15.0 ^a,b^	12.9 ^c,d^	13.6 ^a,b,c,d^	15.2 ^a^	12.3 ^d^	12.3 ^d^	13.2 ^a,b,c,d^
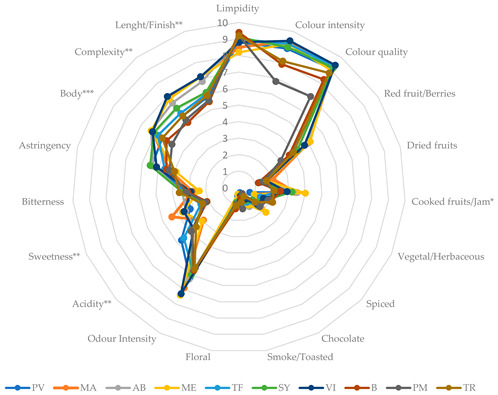										

* Significant (*p* < 0.05); ** Very significant (*p* < 0.01); *** Highly significant (*p* < 0.001). ^a,b,c,d^—classes determined by the post-hoc differential test of Bonferroni at a *p* < 0.05.

**Table 4:** Odor activity values of the odor active compounds in the monovarietal wine samples [‘Petit Verdot’ (PV), ‘Touriga Franca’ (TF), ‘Preto Martinho’ (PM), ‘Bobal’ (B), ‘Marselan’ (MA), ‘Alicante Bouschet’ (AB), ‘Syrah’ (SY), ‘Trincadeira’ (TR), ‘Merlot’ (ME), and Vinhão‘Vinhão’ (VI)].

Chemical Species	Id ^a^	Compound Name	Odor Series	Monovarietal Red Wines									
				PV	MA	AB	ME	SY	TF	VI	B	PM	TR
*Acids*													
	A4	Butanoic acid	Fatty	0.9	1.3	2.1	1.2	2.6	2.0	1.2	1.4	2.1	1.3
	A5	Isovaleric acid	Pastry	85.7	99.2	93.7	70.7	31	97.6	98.4	47.7	59	55
	A6	Octanoic acid	Roasting	3.5	2.6	3.6	2.9	4.2	4.0	3.7	6.0	3.7	3.5
*Alcohols*													
	AL2	Isobutanol	Roasting	1.7	1.4	2.1	1.1	2.2	1.8	0.9	3.1	3.0	1.5
	AL3	Isoamyl alcohols	Fruity	21.6	18.7	24.6	21.1	39.3	26.6	15.2	47.8	39.7	23.2
	AL5	3-Ethoxy-1-propanol	Sweet	1.7	1.0	1.4	0.9	2.1	0.6	0.4	2.0	1.0	0.7
	AL9	3-Mercaptohexanol	Fatty	216.7	216.7	466.7	200	833.3	433.3	283.3	483.3	583.3	350
	AL11	2-Phenylethanol	Floral	21.6	18.1	19.8	21.9	35.1	23.2	23.1	34.2	32.6	19.2
*Aldehydes*													
	AD1	Hexanal ^b^	Herbaceous	0.0	0.0	0.0	1.8	4.0	5.3	2.0	6.2	10.4	4.9
	AD2	(Z)-3-Hexenal ^b^	Herbaceous	132.0	40.0	56.0	28.0	60.0	48.0	0.0	0.0	172.0	60.0
	AD3	Methional	Pungent	184.0	268.0	390.0	252.0	434.0	304.0	274.0	374.0	274.0	190.0
*Esters*													
	E1	Ethyl propanoate	Sweet	2.5	2.4	2.7	2.4	3.2	2.6	1.8	4.2	3.9	2.1
	E2	Ethyl isobutyrate	Fruity	95.9	85.9	85.0	74.5	74.9	70.3	68.9	94.9	108.9	58.3
	E4	Ethyl butyrate	Fruity	13.9	2.0	2.1	4.1	7.6	5.3	1.0	2.1	0-0	2.9
	E5	Ethyl 2-methylbutyrate	Fruity	3.2	2.7	2.3	3.0	1.9	2.2	4.2	2.9	5.4	1.9
	E6	Ethyl isovalerate	Sweet	37.0	24.3	31.3	29.3	12.0	28.7	41.3	45.7	43.0	23.7
	E7	Isoamyl acetate	Chemical	51.4	35.6	57.7	50.3	84.3	158.9	78.9	156.2	81.2	146.2
	E9	Ethyl hexanoate	Roasting	111.0	91.0	115.0	105.2	139.4	145.2	109.4	151.6	115.6	99.2
	E10	Ethyl octanoate	Roasting	232.5	142.5	247.5	192.0	260.5	297.5	195.0	339.0	212.5	214.5
	E13	Phenethyl acetate	Spicy	1.0	0.7	0.8	0.8	1.3	1.9	1.4	2.9	1.3	2.0
*Ketones*													
	K1	2,3-Butanedione	Sweet	7.7	5.3	8.0	6.7	15.4	17.8	6.6	11.4	10.9	14.9
	K2	2,3-Pentanedione	Sweet	3.0	1.3	3.3	6.9	11.8	18.9	6.3	7.3	5.8	11.5
*Phenols*													
	Ph1	Eugenol	Spicy	44.2	91.5	49.3	51.3	26.3	41.7	73.7	65.0	42.2	47
*Pyrazines*													
	Py1	2,3-Diethyl-5-methylpyrazine ^b^	Pungent	1.7	0.0	4.0	0.0	4.7	1.3	0.6	0.0	0.0	0.9
	Py2	2-Ethyl-3,5-dimethylpyrazine ^b^	Herbaceous	4.0	10.7	8.5	3.9	0.0	3.9	1.6	6.3	4.0	2.8

^a^ Identification letters; ^b^ Compound not confirmed.

## Data Availability

The data presented in this study are available within the article.

## Supplementary Materials

The following supporting information can be downloaded at: https://www.mdpi.com/article/10.3390/plants12102063/s1, Table S1: Identification and detection frequency of the pOAC in the monovarietal wine samples analyzed in the GC-O system.
