# Supplementary material for: First Approach to the Aroma Characterization of Monovarietal Red Wines Produced from Varieties Better Adapted to Abiotic Stresses
Source: Plants (Basel). 2023 May 22;12(10):2063. doi: 10.3390/plants12102063 (PMC10224026; doi:10.3390/plants12102063)
Supplement: Supplementary file 1 [file plants-12-02063-s001.zip › plants-2369760-supplementary.pdf]

Supplementary table S1. Identification and detection frequency of the pOAC in the monovarietal wine samples analyzed in the GC-O system.

Chemical species	Id[a]	KI[b]	Compound / CAS Registry Number	Odour descriptor	Odour series	Monovarietal red wines									
						Petit Verdot	Marselan	Alicante Bouschet	Merlot	Touriga Franca	Syrah	Vinhão	Bobal	Preto Martinho	Trincadeira
Acids															
	A1	1454	Acetic acid / 64-19-7	Vinegar, floral, pungent, herbaceous	Pungent	5	7	nd	5	nd	3	3	nd	3	nd
	A2	1551	Propanoic acid / 79-09-4	Vinegar, flowery, soap	Herbaceous	nd	nd	nd	3	nd	nd	nd	nd	nd	nd
	A3	1592	Isobutyric acid / 79-31-2	Sweet, fruity, fresh	Fruity	nd	nd	3	nd	nd	nd	nd	nd	nd	3
	A4	1670	Butanoic acid / 107-92-6	Oily, not good	Fatty	3	nd	nd	nd	nd	nd	nd	nd	nd	nd
	A5	1692	Isovaleric acid/ 503-74-2	Toast, brioche, pastry	Pastry	nd	nd	nd	nd	nd	3	nd	nd	nd	nd
	A6	2097	Octanoic acid / 124-07-2	Toasted bread	Roasting	3	nd	nd	nd	nd	nd	3	nd	nd	nd
Alcohols															
	AL1	1051	1-Propanol / 71-23-8	Fruity, alcohol, sweet	Sweet	4	nd	nd	nd	nd	nd	4	nd	nd	nd
	AL2	1190	Isobutanol / 78-83-1	Cocoa, coffee, banana, cooked potatoes	Roasting	nd	nd	nd	5	3	6	4	6	nd	5
	AL3	1232	Isoamyl alcohols/ 123-51-3 + 137-32-6	Cocoa, banana, chocolate, pungent, oily	Fruity	4	6	8	6	9	9	9	9	8	7
	AL4	1335	3-Methyl-1-pentanol / 589-35-5	Chees, herbaceous, sour	Chemical	nd	3	4	nd	nd	nd	nd	nd	nd	nd
	AL5	1392	3-Ethoxy-1-propanol / 111-35-3	Sweet, tropical fruit	Sweet	nd	nd	nd	3	nd	4	nd	nd	nd	nd
	AL6	1558	2,3-Butanediol / 513-85-9	Cooked potatoes, soil dust	Earth	nd	nd	nd	nd	3	nd	nd	nd	nd	nd
	AL7	1608	1,2-Propanediol / 57-55-6	Toast, sweet	Roasting	nd	nd	nd	4	nd	nd	nd	nd	nd	nd
	AL8	1810	1,3-Propanediol / 504-63-2	Green, oily, peppery	Herbaceous	nd	nd	nd	nd	nd	nd	nd	nd	nd	4
	AL9	1864	3-Mercaptohexanol / 51755-83-0	Herbaceous, oily, not good	Fatty	nd	nd	5	nd	nd	nd	nd	nd	3	nd
	AL10	1906	Benzyl alcohol / 100-51-6	Flowery, banana, oily	Floral	4	nd	nd	nd	3	nd	nd	nd	nd	nd
	AL11	1939	2-Phenylethanol / 60-12-8	Floral, tobacco, banana	Floral	nd	3	3	4	nd	nd	nd	nd	nd	nd
	AL12	2340	Glycerol / 56-81-5	Roses, good	Sweet	3	nd	nd	nd	nd	nd	nd	nd	nd	nd
Aldehydes															
	AD1	1080	Hexanal / 1335-39-3 [c]	Herbaceous, grass	Herbaceous	nd	nd	nd	nd	nd	nd	3	4	nd	4
	AD2	1158	(Z)-3-Hexenal / 6789-80-6 [c]	Grass, green	Herbaceous	nd	4	nd	5	9	4	nd	nd	4	6
	AD3	1472	Methional / 3268-49-3	Fruity, sour, boiled vegetables	Pungent	5	nd	nd	nd	nd	nd	nd	nd	nd	nd
Dioxanes															
	D1	1095	2,5-Dimethyl-1,4-dioxane / 15176-21-3 [c]	Green, grass	Herbaceous	nd	3	nd	nd	nd	nd	nd	4	nd	nd
Esters															
	E1	943	Ethyl propanoate / 105-37-3	Fruity, toasted, sweet	Sweet	nd	nd	nd	nd	nd	nd	nd	3	3	nd

Chemical species	Id[a]	KI[b]	Compound / CAS Registry Number	Odour descriptor	Odour series	Monovarietal red wines									
						Petit Verdot	Marselan	Alicante Bouschet	Merlot	Touriga Franca	Syrah	Vinhão	Bobal	Preto Martinho	Trincadeira
	E2	956	Ethyl isobutyrate/ 97-62-1	Fruity, strawberry, lactic	Fruity	5	4	6	7	5	6	5	6	5	6
	E3	1018	Isobutyl acetate / 110-19-0	Fruity, alcohol, oily	Fatty	nd	nd	nd	nd	nd	4	3	nd	4	5
	E4	1042	Ethyl butyrate / 105-54-4	Fruity, strawberry, soil	Fruity	4	nd	nd	3	4	4	nd	nd	nd	6
	E5	1057	Ethyl 2-methylbutyrate / 7452-79-1	Fruity, strawberry, sweet	Fruity	6	4	5	8	5	5	8	4	5	6
	E6	1073	Ethyl isovalerate / 108-64-5	Fruity, rubbish, jam	Sweet	6	6	8	7	3		4	3		3
	E7	1137	Isoamyl acetate /123-92-2	Banana, solvent	Chemical	nd	nd	nd	nd	3	nd	nd	nd	nd	nd
	E8	1190	Ethyl 3-methylpentanoate / 5870-68-8 [c]	Cooked fruit, apple	Sweet	nd	nd	nd	nd	nd	3	nd	nd	nd	nd
	E9	1246	Ethyl hexanoate / 123-66-0	Butter, cocoa powder, fruity	Roasting	nd	4	3	7	nd	nd	nd	nd	nd	nd
	E10	1446	Ethyl octanoate / 106-32-1	Floral, sweet, charred wood	Sweet	nd	nd	nd	nd	nd	nd	nd	nd	nd	4
	E11	1652	Ethyl decanoate / 110-38-3	Floral, sweet, iodine	Floral	nd	nd	nd	4	nd	nd	nd	nd	3	nd
	E12	1823	Ethyl 4-hydroxybutanoate / 999-10-0	Alcohol, fruity	Fruity	nd	nd	3	nd	nd	nd	nd	nd	nd	nd
	E13	1840	Phenethyl acetate / 103-45-7	Wood, spiced	Spiced	nd	nd	nd	nd	nd	nd	nd	3	nd	nd
	E14	1863	Ethyl dodecanoate / 106-33-2	Sweet, peppery, foot smell	Sweet	nd	nd	5	nd	nd	nd	nd	nd	nd	nd
	E15	2403	Ethyl hydrogen succinate / 1070-34-4	Fruity, moss, charred wood	Fruity	3	nd	4	6	5	nd	nd	5	nd	8
Furans															
	F1	1518	Furfural / 98-01-1	Pastry, toast	Pastry	nd	nd	nd	3	nd	nd	nd	nd	nd	nd
Ketones															
	K1	974	2,3-Butanedione / 431-03-8	Toffee, caramel, lactic, butter	Sweet	8	7	8	9	8	9	6	8	7	9
	K2	1068	2,3-Pentanedione / 600-14-16	Caramel, sweet, butter, brown sugar	Sweet	nd	nd	nd	nd	8	nd	nd	nd	3	nd
Phenols															
	Ph1	2190	Eugenol / 97-53-0	Clove, spiced, floral	Spiced	3	nd	nd	nd	nd	nd	nd	nd	nd	nd
Pyrazines															
	Py1	1495	2,3-Diethyl-5-methylpyrazine / 18138-04-	Vinegar, meaty, toast	Pungent	nd	nd	nd	nd	5	nd	nd	nd	nd	nd
	Py2	1497	2-Ethyl-3,5-dimethylpyrazine / 13925-07-	Herbaceous, acetic	Herbaceous	nd	3	nd	nd	nd	nd	nd	nd	nd	nd
Unknown															
	Un1	1480		Vinegar, grass, oily	Pungent	nd	nd	nd	nd	nd	nd	nd	3	nd	nd
	Un2	1488		Metallic, not good, foot smell	Chemical	4	nd	nd	nd	nd	nd	nd	nd	nd	4
	Un3	1729		Smoke, burning	Roasting	nd	nd	nd	3	nd	nd	nd	nd	nd	nd
	Un4	2029		Floral, herbal, sweet	Floral	nd	nd	nd	nd	4	nd	nd	nd	nd	nd
	Un5	2074		Spiced, soap, chocolate	Spiced	nd	nd	nd	nd	3	nd	nd	nd	nd	nd
	Un6	2367		Wet stone, fermenting fruit	Pungent	nd	nd	nd	nd	nd	nd	nd	nd	nd	3

Chemical species	Id[a]	KI[b]	Compound / CAS Registry Number	Odour descriptor	Odour series	Monovarietal red wines									
						Petit Verdot	Marselan	Alicante Bouschet	Merlot	Touriga Franca	Syrah	Vinhão	Bobal	Preto Martinho	Trincadeira

[a] Identification letters; [b] Kovats Ritention Index in DB-WAX GC Column; [c] compound not confirmed; nd – not detected.
